# Psychotherapy via the Internet: What Programs Do Psychotherapists Use, How Well-Informed Do They Feel, and What Are Their Wishes for Continuous Education?

**DOI:** 10.3390/ijerph17218182

**Published:** 2020-11-05

**Authors:** Elke Humer, Peter Stippl, Christoph Pieh, Wolfgang Schimböck, Thomas Probst

**Affiliations:** 1 Department for Psychotherapy and Biopsychosocial Health, Danube University Krems, 3500 Krems, Austria; elke.humer@donau-uni.ac.at (E.H.); christoph.pieh@donau-uni.ac.at (C.P.); 2 Austrian Federal Association for Psychotherapy, 1030 Vienna, Austria; oebvp.stippl@psychotherapie.at (P.S.); wolfgang.schimboeck@liwest.at (W.S.); 3 Viktor Frankl Education Austria (ABILE), 3390 Melk, Austria

**Keywords:** psychotherapy, internet, COVID-19, software, information needs

## Abstract

The outbreak of the COVID-19 pandemic has caused changes in the provision of psychotherapy around the world. The common format of delivering in-person psychotherapy is replaced by psychotherapy via the Internet to a great extent. This study examined how well Austrian psychotherapists feel informed about the use of the Internet in psychotherapy, where additional information needs exist, and which software is used. A link to an online survey was sent to all psychotherapists providing a valid email address in the official list of licensed psychotherapists at the start of the COVID-19 lockdown in Austria. A total of 1547 people took part in the survey. The results show that psychotherapy via the Internet was primarily offered via Skype and Zoom during the COVID-19 pandemic and that the majority of the therapists felt well-informed about psychotherapy via the Internet; however, several therapists stated that they wish to have further information on data protection and security. Overall, the study shows that Austrian psychotherapists coped well with the rapid change from the provision of psychotherapy through personal contact to psychotherapy via the Internet. Security and data protection aspects of therapy via the Internet should be addressed in training and further education of psychotherapists. As this study was conducted online, it might have caused some respondent bias towards a higher participation of psychotherapists with higher preference for new technologies.

## 1. Introduction

The COVID-19 pandemic enhances psychological distress, resulting in an increased need for mental health care [1,2,3]. In the Austrian general population, for example, depressive symptoms, anxiety symptoms and insomnia symptoms were higher during the COVID-19 lockdown than in previous studies before the lockdown [4]. A follow-up investigation of this Austrian sample showed that more individuals changed from not depressed in lockdown to depressed after lockdown (when restrictions had been lifted for some time) as compared to from depressed in lockdown to not depressed after lockdown [5].

The first COVID-19 cases in Austria were confirmed on the 25 February 2020. The Austrian government introduced obligatory COVID-19 lockdown measures on the 16 March 2020, which lasted until end of April 2020. A nationwide curfew entailed restrictions in movement and activities with the following five exceptions: (1) addressing immediate danger, (2) errands to meet basic needs, (3) fulfilling work responsibilities (if working from home is not possible), (4) care and assistance for people in need and (5) outdoor activities (alone or with pets or people from the same household). At least one meter distance between people had to be ensured. During that time, certain areas in Austria were under quarantine and thus faced even more severe restrictions at the time of the study.

Results regarding psychological distress in Austria [4,5] and worldwide [6,7] indicate an increased need for mental health care. Psychotherapy is an established treatment for mental health problems, which traditionally takes place in a face-to-face setting with direct contact. The COVID-19 pandemic requires significant changes in the setting in which psychotherapy is provided [8]. To combat the rapid spread of the virus, reducing direct contact represents one of the most important measures; thus, many therapists switched to alternative psychotherapy formats [9]. In many countries, psychotherapy with personal contact is being largely replaced by psychotherapy via the Internet or telephone [10]. However, a survey among Austrian psychotherapists showed that the increase in distance psychotherapy during the COVID-19 lockdown could not fully compensate for the decrease in psychotherapy with personal contact [11]. Therefore, it is essential to elucidate possible barriers in the implementation of remote psychotherapy. Studies have shown that psychotherapists are in general more skeptical about remote psychotherapy than their patients [12]. In order to ensure sufficient provision of high-quality mental health care during the COVID-19 pandemic, it is important to investigate possible aspects that might support the practice of remote psychotherapy. During the COVID-19 lockdown, most health insurances in Austria started to cover the costs for psychotherapy via telephone and the Internet in the same way as for face-to-face psychotherapy. Yet, psychotherapy via the Internet is rejected by the official Austrian guideline related to the Internet and psychotherapy, which was valid at the time of the COVID-19 situation [13]. Therefore, Austrian psychotherapists are in a difficult position in times of COVID-19. On the one hand, health insurances cover the costs for psychotherapy via the Internet, while on the other hand, the official guidelines reject the Internet for psychotherapy.

Therefore, we focused on remote psychotherapy via the Internet in the present study. The aim of this cross-sectional survey was to evaluate how well psychotherapists in Austria feel informed about the use of the Internet in psychotherapy and in which areas there is an additional need for information. Another aim was to determine which software was used during the COVID-19 lockdown for psychotherapy via Internet.

The following research questions (RQ) were investigated:

RQ1a: How well do psychotherapists feel informed about psychotherapy delivered via the Internet during the first weeks of the COVID-19 lockdown in Austria and is there a difference with respect to age and gender?
RQ1b: Is there a difference in the subjective information level between psychotherapists who already used the Internet for psychotherapy before the COVID-19 pandemic and those with no previous experience?
RQ1c: Is there a difference with regard to how well psychotherapists feel informed about psychotherapy via the Internet between psychotherapists who provided psychotherapy during the COVID-19 lockdown via the Internet and those who did not?
RQ1d: Is there a difference in the subjective information level in therapists who started to treat via the Internet during the COVID-19 lockdown as compared to those who did not newly start with therapy via the Internet during the COVID-19 lockdown?
RQ2a: How high is the wish of psychotherapists for additional information about psychotherapy provided via the Internet and is there a difference with respect to age and gender?
RQ2b: Is there a difference in the subjective need for further information between psychotherapists who already used Internet for psychotherapy before the COVID-19 pandemic and those with no previous experience?
RQ2c: Is there a difference regarding the wish for further information about psychotherapy via the Internet between psychotherapists who provided psychotherapy during the COVID-19 lockdown via the Internet and those who did not?
RQ2d: Is there a difference in the subjective need for further information about psychotherapy provided via the Internet in therapists who started to treat patients via the Internet during the COVID-19 lockdown as compared to those who did not newly start with therapy via the Internet during the COVID-19 lockdown?
RQ3: In which areas related to psychotherapy via the Internet is there an additional need for information in psychotherapists during the COVID-19 lockdown?
RQ4: Which software was used for psychotherapy via the Internet during the COVID-19 lockdown in Austria?

## 2. Materials and Methods 

### 2.1. Study Design

An online survey comprising 79 questions in total was created using ResearchElectronicDataCapture (REDCap) [14]. A link to the survey was sent to all psychotherapists registered in the official list of the Federal Ministry for Social Affairs, Health, Care and Consumer Protection (BMSGPK) who had provided a valid email address (approximately 6000 psychotherapists out of more than 9000 licensed psychotherapists in March 2020). The online survey was editable from 24 March–1 April 2020. More details about the survey can be found in the other publications related to this survey [11,15,16,17]. The methods were approved by the ethics committee of Danube University Krems.

### 2.2. Variables

The following questions were analyzed in the current study:

Psychotherapists in Austria were asked about their age, gender, years in profession, therapeutic orientation, and number of patients treated on average per week via the Internet since the COVID-19 lockdown as well as (retrospectively) in the months before the lockdown. For the statistical analyses, these numbers were set to 0 for the participating psychotherapists stating that they did not practice psychotherapy via the Internet in the months before/during the COVID-19 lockdown. 

Psychotherapists were asked how well-informed they currently feel about the use of the Internet in psychotherapy. Specifically, this question was related to the official Austrian Internet guideline for psychotherapists [13], the information provided by the Ministry of Health regarding the current situation regarding COVID-19, and an update by the Austrian Federal Association for Psychotherapy (ÖBVP) on the current situation regarding COVID-19. Participants were asked to rate their own level of information on a sliding scale from 0 (“not at all”) to 100 (“very good”).

Another question addressed whether therapists wish to have better information regarding the use of Internet in psychotherapy. Again, psychotherapists were asked to rate on a sliding scale from 0 (“does not apply at all”) to 100 (“applies completely”).

Thereafter, therapists who stated that they wish to have better information regarding the use of the Internet in psychotherapy were asked to describe what specific information they would like in a free text question.

A final free text question raised which Internet programs are used to treat patients psychotherapeutically.

### 2.3. Data Analyses

The closed questions were analyzed using SPSS version 26 (Inc., Chicago, IL, USA). 

Descriptive statistics were conducted to describe the demographic characteristics and scales mean values. 

To address RQ1 and RQ2, *t*-tests for independent samples as well as Pearson correlation coefficients (r) were applied and *p*-values of less than 0.05 were considered statistically significant (2-sided tests). As an effect size measure, Cohen’s d was calculated, which can be interpreted as follows: small effect 0.2–0.5, medium effect 0.5–0.8, large effect >0.8. 

To answer RQ3 and RQ4, the two free text questions were evaluated graphically using word clouds (WordClouds, Zygomatic, Vianen, The Netherlands). The answers to the question about specific information needs were translated from German to English using Google Translate (Google LLC, Mountain View, CA, USA).

## 3. Results

### 3.1. Sample Description

A total of 1547 psychotherapists took part in the online survey, which corresponds to a response rate of around 25%. Psychotherapists were, on average, 51.7 years old (standard deviation (SD) = 9.7 years). The proportion of female participants was 75.7%, which corresponds to the total population of Austrian psychotherapists, with a total of 74.1% of female therapists in the official Austrian list of licensed psychotherapists (March 2020). On average, the professional experience was 11.2 (SD = 9.2) years. 

The distribution of the psychotherapeutic orientations compared to the distribution of the therapeutic orientations in the list of the BMSGPK (March 2020) was as follows:

- Psychodynamic: 20.9% of the participants vs. 25.9% in the list of the BMSGPK
- Humanistic: 46.3% of the participants vs. 37.8% in the list of the BMSGPK
- Systemic: 22.0% of the participants vs. 24.3% in the list of the BMSGPK
- Behavioral therapy: 9.8% of the participants vs. 12.0% in the list of the BMSGPK.

The therapeutic orientation of 1% of the sample could not be further specified. Psychotherapists with a humanistic focus were therefore overrepresented in the sample.

### 3.2. Information about Psychotherapy via Internet (RQ1)

- Results for RQ1a:

When asked how well-informed therapists currently feel about the use of the Internet in psychotherapy, the 1547 participating therapists gave an average value of 75.95 (SD = 23.23), with the answers ranging from 0 to 100. Female psychotherapists rated their subjective information level higher (M = 76.69, SD = 22.60) than their male colleagues (M = 73.65, SD = 24.96), T (585.5) = 2.100, *p* = 0.036. However, the effect size was very small, with Cohen’s d = 0.131, 95% CI: 0.015, 0.247. No age effect was observed (r = < 0.001, *p* = 0.998).

- Results for RQ1b:

The subjective information level was not affected by previous experience with psychotherapy via the Internet. The 122 psychotherapists who already used the Internet in psychotherapy before COVID-19 rated their subjective information level (M = 75.66, SD = 25.25) similarly to those 1425 psychotherapists who did not use the Internet before COVID-19 for psychotherapy (M = 75.97, SD = 23.05), t (1545) = −0.141, *p* = 0.888.

- Results for RQ1c:

The subjective information level differed with respect to the format psychotherapy was provided during the first weeks of the COVID-19 lockdown (t (1297.2) = −2.158; *p* = 0.031. Therapists not treating via the Internet during the first weeks of the COVID-19 lockdown (*n* = 639) felt less informed (M = 74.41, SD = 24.36) than the 908 psychotherapists treating via the Internet (M = 77.03, SD = 22.34). The effect size was very small, with Cohen’s d = −0.113, 95% CI: −0.214, −0.012.

- Results for RQ1d:

An analysis of psychotherapists who newly started with Internet psychotherapy during the lockdown (*n* = 792) as compared to the remaining psychotherapists (*n* = 755) revealed that new starters felt better informed (M = 77.23, SD = 21.90) than the rest (M = 74.60, SD = 24.48), t (1507.3) = 2.223, *p* = 0.026. The effect size was very small, with Cohen’s d = 0.113, 95% CI: 0.014, 0.213.

### 3.3. Need for More Information Regarding the Use of Internet in Psychotherapy (RQ2)

- Results for RQ2a:

When asked whether therapists would wish to receive better information regarding the use of the Internet in psychotherapy, the average of a total of 1547 therapists stated a value of 42.56 (SD = 32.35). Ratings ranged again from 0 to 100. No differences between female (M = 42.66, SD = 32.44) and male (M = 42.25, SD = 32.11) therapists emerged (T (1545) = 0.212, *p* = 0.832). A significant negative correlation with age was observed (r = −0.074, *p* = 0.004), indicating a decrease in the wish for better information with increasing age; however, the Pearson correlation coefficient was very low.

- Results for RQ2b:

The wish for further information about Internet psychotherapy was affected by previous experience with psychotherapy via the Internet. Psychotherapists who already used the Internet in psychotherapy before COVID-19 (*n* = 122) had a higher wish for additional information (M = 48.55, SD = 32.83) than those who did not use the Internet before (*n* = 1425, M = 42.05, SD = 32.27), t (1545) = 2.133, *p* = 0.033. The effect size was small, with Cohen’s d = 0.201, 95% CI: 0.016, 0.386.

- Results for RQ2c:

The wish for further information differed with respect to the format in which psychotherapy was provided during the first weeks of the COVID-19 lockdown (t (1545) = −4.879; *p* < 0.001. Therapists not treating via the Internet during the first weeks of the COVID-19 lockdown (*n* = 639) wished for less additional information (M = 37.81, SD = 32.01) than psychotherapists treating via Internet (*n* = 908, M = 45.90, SD = 32.19). The effect size was small, with Cohen’s d = −0.252, 95% CI: −0.353, −0.150.

- Results for RQ2d:

An analysis of psychotherapists who started with Internet psychotherapy during the lockdown (*n* = 792) as compared to the remaining psychotherapists (*n* = 755) revealed that new starters wished for more information (M = 45.49, SD = 32.15) than the rest (M = 39.49, SD = 32.30), t (1545) = 3.658, *p* < 0.001. The effect size was very small, with Cohen’s d = 0.186, 95% CI: 0.086, 0.286.

### 3.4. Specific Information Needs Regarding the Use of Internet in Psychotherapy (RQ3)

In response to the free text question, psychotherapists who stated that they want better information regarding the use of the Internet should be provided with specific information. A total of 647 therapists gave a valid answer to the free text question about specific information needs (excluding answers such as “none” or “0”, “x” etc.). These therapists rated, on average, 66.45 (SD = 26.70) on the closed question about the need for better information, and thus differed significantly from the 900 therapists who did not answer the free text question (M = 25.39, SD = 24.12; t (1545) = 31.58; *p* <.001). The effect size was high, with Cohen’s d = 1.627, 95% CI: 1.511, 1.744.

**Figure 1:**
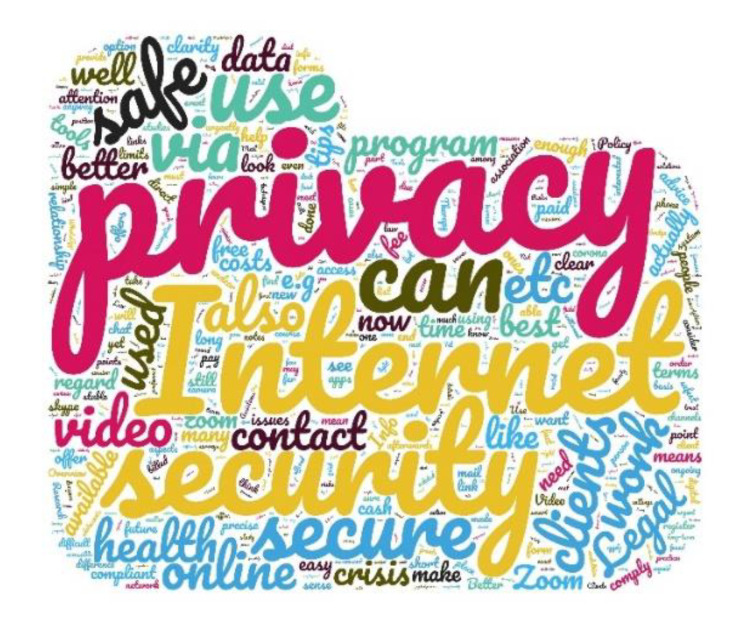
Word cloud on the answers to the free text question about which topics psychotherapists would like more information.

In order to get an overview of the specific information needs of psychotherapists regarding additional information about psychotherapy via the Internet, Figure 1 depicts a word cloud that results from the free text answers. A larger number of words is indicated by a larger font. It can be seen that there is an additional need for information, particularly in the areas of data protection (translated from German to English as “privacy”) and security.

### 3.5. Software Used (RQ4)

**Figure 2:**
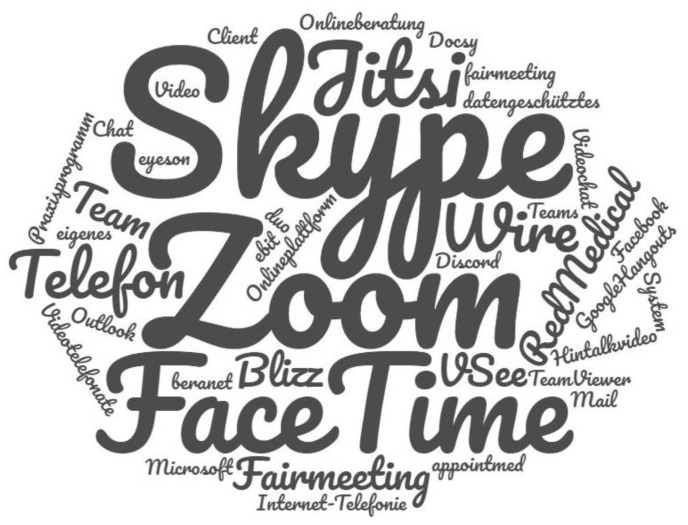
Word cloud on the answers to the free text question about the software used for psychotherapy via Internet.

A free text question raised which software was used to conduct psychotherapy via Internet. Figure 2 illustrates the information provided by a total of 883 therapists who gave a valid answer in the form of a word cloud. Skype ranked first (563 responses), followed by Zoom (317 responses), TheraPsy (113 responses), WhatsApp (77 responses), FaceTime (73 responses) and Signal (65 responses). The programs ClickDoc, Jitsi, Instahelp, Webex, Wire and Whereby were mentioned 5 to 15 times.

## 4. Discussion

The results show that the majority of therapists felt well-informed about psychotherapy via the Internet during the first weeks of the COVID-19 lockdown in Austria. In particular, female psychotherapists, psychotherapists treating patients via the Internet during the first weeks of the COVID-19 lockdown, and psychotherapists who newly started to use Internet for psychotherapy in the COVID-19 lockdown stated that they feel well-informed about psychotherapy via the Internet. Additional information needs exist with regard to data protection aspects and security. Psychotherapy via the Internet was carried out primarily via Skype and Zoom during the COVID-19 lockdown.

This study suggests that Austrian psychotherapists coped well with the rapid change from the provision of psychotherapy with personal contact to psychotherapy via the Internet. This is in agreement with our recent finding that subjective stress-level as well as job-related worries and fears of existence were not affected by the format in which psychotherapy was provided during the COVID-19 lockdown in Austria [17]. More specifically, no difference was observed between psychotherapists treating only face-to-face, face-to-face as well as via telephone or the Internet, only via telephone or Internet, or not at all in the first weeks of the COVID-19 lockdown. In addition, stress-level and job anxiety did not differ between psychotherapists who already used a telephone or the Internet for psychotherapy in the months before COVID-19 and those who did not use a telephone or the Internet for psychotherapy in the months before COVID-19 [17]. The majority of therapists reported that experiences with psychotherapy via the Internet was better than previously expected [16]. 

The official Internet guideline for psychotherapists in Austria rejects psychotherapy via the Internet [13]. During the COVID-19 lockdown, however, health insurance companies started to reimburse or partially reimburse the costs of psychotherapy via the Internet. Overall, it would therefore not be surprising if psychotherapists felt insufficiently informed due to this contradiction as to whether the practice of psychotherapy via the Internet or telephone is lege artis during the COVID-19 pandemic or not. The majority of the therapists, however, indicated that they felt adequately informed, and analysis of the closed question also shows a moderate desire for additional information. The relatively high number of therapists (around 42% of the total participants) who provided valid information in response to the free text question regarding further information needs, however, makes it clear that in practice, more information is currently required, especially in terms of data protection and security. Concerns about security and data protection for online therapies have also been reported in previous studies [12,18].

Skype was clearly the most common software used. One reason could be that Skype was the first software that enabled video phone calls over the Internet for free and has been available since 2003. Therefore, many therapists may have been familiar with this software before the COVID-19 pandemic. Next to Skype, over 300 therapists also used Zoom for providing psychotherapy. One reason for the lower use of Zoom could be that Zoom represents a newer software (available on the market since 2011). Regardless of the chosen software, data protection issues seem to play a major role in psychotherapy via the Internet in general [19], which is also confirmed by the results of the current study. 

Overall, it should be kept in mind that this study may not provide a representative picture of Internet psychotherapy during the COVID-19 pandemic. As the survey was conducted online, it is possible that this survey primarily involved psychotherapists who were already more open to online therapy before the pandemic or those who had a greater affinity for digital media, respectively. The study also represents a snapshot. Further studies should evaluate the usage of psychotherapy via the Internet when lockdown measures were lifted, and also whether long-term changes—beyond the period of the pandemic—occur. 

## 5. Conclusions

Overall, it can be concluded that the COVID-19 pandemic led to an increased use of the Internet in psychotherapy. In particular, in terms of data protection and security, additional information is required so that psychotherapists feel more confident in the provision of psychotherapy via Internet during but also—if it will be allowed in Austria—after the COVID-19 pandemic. It would be advisable to consider psychotherapy from a distance in the training of psychotherapists, since psychotherapy from a distance can bring benefits not only in acute crises, but also in other situations. Although remote psychotherapy can and should not completely replace face-to-face psychotherapy, it improves access to mental health care services to patients facing logistic or stigma-related barriers to receiving face-to-face psychotherapy [20]. Studies have also shown comparable effectiveness of providing psychotherapy via the Internet as compared to psychotherapy with personal contact [21,22,23]. Thus, remote psychotherapy should be considered as valuable and legal option for mental health care in the future.
